# Parliament and the Profession

**Published:** 1916-01-15

**Authors:** 


### PARLIAMENT AND THE PROFESSION.

### House of Commons Proceedings.

SINCE Parliament reassembled after the Christmas recess little business of any particular note affecting the interests of the medical profession has been transacted, and few questions relating to medical affairs have been asked. Even the small coterie of members who frequently raise points in connection with National Health Insurance affairs have been almost silent. Statements have been made concerning the health of the troops both at home and abroad, and the value of anti-typhoid inoculation was strikingly demonstrated by statistics furnished from France and Belgium.

### Sickness on Salisbury Plain.

REPLYING to Sir E. Cornwall, Mr. Tennant stated that the annual ratios of sickness and mortality per thousand troops were, for the Salisbury Plain district, during the period September 1 to December 31: Admissions, 325.4; deaths, 1.88. Both these ratios are lower than those for peace-time, and under the circumstances the War Office cannot see any necessity for the appointment of a Committee to consider the question of the health of the troops in this district.

### Treatment of Insured Consumptives in Edinburgh.

THE Town Council of Edinburgh recently issued a report in which the wisdom of continuing the present scheme for the sanatorium treatment of phthisis patients was called in question on the ground that the current expenditure in the city of £19,000 a year on sanatorium treatment was not calculated to attain its object, and was therefore described as futile. A copy of this report has been received by the Chancellor of the Exchequer, and the subject is receiving the consideration of the Retrenchment Committee.

### The Value of Inoculation Against Typhoid.

REPLYING to Mr. W. Thorne, Mr. Tennant stated that from the beginning of hostilities to November 10, 1915, 1,365 cases of enteric fever were reported as occurring among the British troops in France and Belgium. Of those who had been definitely diagnosed after bacteriological examination there were 579 cases where there had been inoculation, among which there were thirty-one deaths. Of 571 cases where there had been no inoculation there were 115 deaths. A comparison of these figures with the records of disease of other wars affords convincing evidence of the infinite value of inoculation. In the United Kingdom from August 1, 1914, to October 30, 1915, 540 cases of enteric fever were reported and eighty-seven deaths; 39 per cent. of these cases occurred in men who had not been inoculated, but Mr. Tennant was unable to say how the deaths were distributed among the inoculated and uninoculated respectively. For paratyphoid no system of inoculation has yet been adopted.

### Female Nurses in Mental Hospitals.

THE attention of the Home Office has again been directed to the question of the desirability, or otherwise, of employing female nurses in mental hospitals. Among the institutions affected is the Wakefield Asylum, where female nurses are being employed in four of the twenty-five wards for male patients. But male attendants are also being employed in these wards for certain duties, and the inmates are all quiet cases which, in the view of the Board of Control, can properly be attended by women. Mr. O'Grady is anxious that instructions should be given that no further attendants shall be recruited to His Majesty's Services until asylum authorities are in a position to replace them by temporary male labour, but Mr. Brace, who replied to the question, asked on this point, intimated that the matter was being carefully watched by the Board of Control, and that the employment of female nurses to release men for enlistment would continue to be allowed in suitable cases and under proper safeguards.

### Lunacy Institutions and Wounded Soldiers.

REPLYING to a question asked by Mr. King, the Under-Secretary of State for the Home Department made a statement with regard to the number of institutions under the Lunacy Commissioners which have been utilised for the accommodation of sick and wounded soldiers. Ten asylums in England and Wales and isolated parts of two others have been placed at the disposal of the War Office for use as hospitals, and accommodation has thereby been provided for over 14,000 men. Arrangements are in contemplation for converting into hospitals two more asylums. In addition, an asylum which at the outbreak of war was nearly completed but not yet occupied was taken over by the War Office, and a similar course is to be adopted with another new asylum now approaching completion. Nearly all the civilian patients displaced by these arrangements have been distributed over the remaining asylums, with the result that most of these asylums have at present more inmates than would be considered advisable by the Board of Control in normal times. Every effort, however, is being made to minimise the inconvenience of the arrangement, which is only justified by the exceptional circumstances of the present time.

